# Classroom network structure learning engagement and parallel temporal attention LSTM based knowledge tracing

**DOI:** 10.1371/journal.pone.0320303

**Published:** 2025-04-07

**Authors:** Zhaoyu Shou, Yihong Li, Dongxu Li, Jianwen Mo, Huibing Zhang

**Affiliations:** 1 School of Information and Communication, Guilin University of Electronic Technology, Guilin, China; 2 Guangxi Wireless Broadband Communication and Signal Processing Key Laboratory, Guilin University of Electronic Technology, Guilin, China; 3 School of Computer and Information Security, Guilin University of Electronic Technology, Guilin, China; Zhejiang Normal University, CHINA

## Abstract

In order to accurately assess the students’ learning process and the cognitive state of knowledge points in smart classroom. A classroom network structure learning engagement and parallel temporal attention LSTM based knowledge tracing model (CL-PTKT) is proposed in this paper. First, a classroom network is constructed based on the information of student ID, seating relationship, and head-up/head-down state obtained from the smart classroom video. Second, a learning engagement model is established by utilizing the head-up/head-down state of students and the structural characteristics of the classroom network. Finally, in this paper innovatively proposes a parallel temporal attention LSTM feature tracking algorithm based on the learning engagement model and the knowledge-exercise data. It can fully considers the potential associated attributes of the knowledge-knowledge, knowledge-exercise and knowledge-learning engagement. And accurately characterizes the knowledge state during the knowledge point’s lecture time. To provide effective support for teachers to make seat adjustments and accurate interventions for in the teaching process. This paper conducts extensive experiments under four real datasets. The algorithm in this paper shows optimal performance in all four evaluation metrics compared to the state-of-the-art knowledge tracing models.

## Introduction

Improving learning effect is the eternal theme of education, and measuring learning outcomes can be analyzed in terms of cognitive engagement, affective engagement, and behavioral engagement. In the classification of educational objectives, Bloom separates cognition into a module, which indicates that cognition has an important place in education. Meanwhile, the research on quantitatively assessing learners’ knowledge of knowledge points is becoming increasingly important in the field of smart education [1]. A large number of researchers have analyzed the cognitive state of students in terms of the knowledge tracing model. Masaki Uto et al. [2] proposed to identify the extent of students’ knowledge based on their answers of questions with certain characteristics such as difficulty and differentiation. This study provides new ideas for cognitive assessment of students. To address the problem of inadequate extraction of dependency features of knowledge points in knowledge tracing models, Haowen Yang et al. [3] proposed a novel quantitative relationship neural network for explainable cognitive diagnosis model (QRCDM). It utilizes explicit and implicit correlations between exercises and relating knowledge concepts to calculate students’ mistakes and guesses. In this way, it improves the utilization of attribute relations and enhances the explainability of the model. For the lack of contextual feature extraction for exercise-response in existing models. Cui et al. [4] proposed a multi-relational transformer for knowledge tracing (MRT-KT). Modeling fine-grained interactions between exercises and responses.

However, most existing knowledge tracking models are limited to analyzing students’ knowledge state based on their historical test information on online education platforms. The analysis was based primarily on student test data. At the same time, oﬄine teaching methods are still the dominant mode of education at present. In a smart classroom, students’ cognitive states are influenced by multiple and complex factors. These factors include learning engagement in the learning process, interaction and cooperation among students [5]. Liu et al. [6] suggested that there is an interactive relationship between affective and cognitive inputs, which is important for predicting academic performance. And students’ affective engagement can be mapped to students’ performance of learning behaviors in the classroom. Therefore, existing knowledge tracing models when applied to complex smart classroom environments, it will expose the limitations of its single-dimensional data analysis. Therefore, taking into account the characteristics of students’ learning engagement in smart classroom, it can characterize students’ cognitive state more accurately. And can be better understanding and promote of students’ learning processes.

The learning engagement reflects the degree of attention and participation in learning tasks by students. It reflects the learning state of students to a certain extent [7]. Delgado et al. [8] used deep convolutional neural networks and head pose estimation methods to analyze students’ attentional state (pitch and yaw angle). To obtain students’ learning engagement. Meanwhile, students are easily influenced by the students around them during classroom learning. And this implicit impact is often reflected in students’ classroom learning status and academic performance. Yang et al. [9] used questionnaires and case study methodology, It was shown through a two-way repeated measures analysis of variance (ANOVA) that adjusting students’ seating affects students’ learning engagement in the classroom. Putnik et al. [10] found a significant correlation between students’ academic performance and social network structural characteristics. By analyzing mutual support relationships between students, van Rijsewijk et al.[11] found an association between academic achievement and an unbalanced distribution of classroom helping relationships. Gutierrez et al.[12] found that effective seating arrangements could promote social interaction and classroom participation. And thus improved student learning outcomes. Li et al.[13] proposed a model of emotional transmission determined by students’ facial expressions of one kind. Experimental analysis showed the existence of learning emotions transmitted from student to student in classroom networks. This would affected the individual’s learning.

To address the difficulty of existing knowledge tracing models to accurately characterize students’ knowledge state in complex smart classroom. This paper is based on classroom network structure learning engagement and knowledge point test data. A knowledge tracing model based on classroom network structure learning engagement and parallel temporal attention LSTM (CL-PTKT). The parallel temporal attention LSTM algorithm is used for feature tracking. It can fully consider the potential associated attributes of the knowledge–knowledge, knowledge–exercise and knowledge–learning engagement. To achieve accurate characterization of learners’ cognitive state under smart classroom.

This paper consists of the following main contributions:

1. A learning engagement model utilizing students’ head-up/head-down state and the classroom network structure characteristics is proposed.
2. A parallel temporal attention LSTM feature tracking algorithm is proposed, which integrates classroom network structure learning engagement and knowledge–exercise data to analyze cognitive state so that the limitations of single-dimensional analysis can be avoided. Meanwhile, the extraction of key feature information is enhanced by the temporal attention mechanism, which can characterize the learner’s cognitive state more accurately.
3. A large number of experiments were carried out on four real datasets, and the experimental results proved that the CL-PTKT has certain advantages over the state-of-the-art knowledge tracing algorithms.

The rest of the paper unfolds as follows: The next section, “Related Works” delves into knowledge tracing model. The “Relevant definitions” section then explains the relevant definitions of the algorithms in this paper. ”CL-PTKT model” describes the proposed method in detail. ”Experimental results and analysis” shows the experimental results and reliability of the algorithm in this paper. Finally, this paper summarizes in the ”Conclusions and future work” section and highlight the future research directions.

## Related work

The existing knowledge tracing models fall into two categories: statistics-based and deep learning-based. The more classical statistical knowledge tracing model is the BKT-based model proposed by Corbett et al. [14–16], which modeled a learner’s potential knowledge state as a set of binary variables that represent whether or not a knowledge point is grasped. Liu et al. [17] hypothesized that as learners master more number of knowledge points. The higher the probability that it will be answered correctly. Statistical-based knowledge tracing models can make the prediction results interpretable. However, they couldn’t accurately express the knowledge competencies corresponding to multiple knowledge points when interpreting them. And couldn’t dynamically assess students’ knowledge competencies. Therefore, in order to track students’ knowledge ability in real time, this paper explores deep learning models with powerful fitting capabilities based on big data of classroom students’ behaviors.

To address the limitations brought about by traditional methods. And to adequately deal with the complex relationship of nonlinear interaction between learners and exercises. A large number of researchers had devoted themselves to the study of deep learning-based knowledge tracing models. Piech et al. [18] proposed a DKT model with Recurrent Neural Networks (RNN) as the main body for knowledge tracking of students. It proved for the first time the effectiveness and potential of deep learning in knowledge tracing tasks. And solved the problem that the traditional model could not learn the features of knowledge relations autonomously. Zhang et al. [19] attempted to apply Dynamic Key-Value Pair Memory Networks (DKVMN) to knowledge tracking. It integrated knowledge relationships to obtain more accurately predict knowledge state prediction than the DKT model.

In order to address the problem of forgetfulness that exists in students. Im et al. [20] reacted the Forgetting-aware Linear Bias (FoLiBi) and applied it to a knowledge tracing model based on a contrastive learning framework(CL4KT). Meanwhile, Abdelrahman et al. [21] summarized that sequence modeling KT models could fully exploit the correlation features between test data. It improved prediction accuracy to some extent. However, they also suggested that knowledge tracing algorithm decisions should take into account the correlation with students’ classroom behavioral performance, learning engagement, and curriculum design. Therefore, such methods analyzed knowledge state with single data and lack of consideration of other factors affecting students’ knowledge state. It would decrease the interpretability of the model.

To address the weak ability of existing models to model knowledge point relationships. A large number of researchers had applied attentional mechanisms to knowledge tracing models. Pandey et al. [22] were the first to apply attentional mechanisms to knowledge tracing model. It determined the importance of different historical interactions to current student knowledge state by the scaled dot-product attention mechanism. Ghosh et al. [23] proposed attentive knowledge tracing model (AKT). The attention weights were computed using exponential decay and a context-aware relative distance measure. Choi et al. [24] categorized a series of students’ interactions into question and response embedding sequences based on the students. To address the problem that existing KT models ignore the existence of discrepancies between exercises and prior knowledge. Mao et al. [25] proposed a fine-grained knowledge tracing model (FGKT). It captured correlations between exercises and historical interactions by designing an effective attention mechanism. Huang et al. [26] proposed to increase the time consumed by the student testing process. And proposed a time-distance attention mechanism to predict learners’ knowledge state. Attentive KT model predicted a student’s current state by assigning different weights to different historical interactions. It improved interpretability. However, such methods were mainly based on a larger model of the attention mechanism constructed by encoders and decoders. It required a lot of data training to get better results. It is not applicable to small datasets in smart classrooms.

Meanwhile, in order to incorporate more features to accurately characterize students’ knowledge state. Wang et al. [27] proposed to obtain low-dimensional embeddings for channels with different features using stacked autoencoders. To strengthen the intrinsic links between learning characteristics. Kim et al. [28] reconstructed input features in conjunction with the difficulty of knowledge test questions. And to explore the multiple facets. Liu et al. [29] explored students’ practice records and the text content of the corresponding exercises. They proposed a general Exercise-Enhanced Recurrent Neural Network (EERNN) framework. And extends EERNN into an interpretable exercise-aware knowledge trace (EKT) in conjunction with the concept of knowledge. To predict students’ knowledge state. Yin et al. [30] used a masked language model (MLM) for a pre-training task to learn the representation of practice texts. It improved the performance of the EERNN model further. Text-Aware KT models made some headway in the field of knowledge tracing. However, because of the lack of consistency between datasets. And text markup is difficult. It is difficult to apply it widely.

In recent years, the continuous development of graph neural networks. Graph neural networks can adaptively extract spatial information between data. It is automatically updated by edge weights to obtain the node state. Shun Mao et al [31] proposed improved graph attention networks (GATs). It aggregated the knowledge structure features between knowledge concepts and test questions to obtain valid input. Su et al [32] proposed to incorporate features of the test and knowledge concepts. It solved the problem of multiple knowledge points and gained more predictive performance. To address test data sparsity and multiple knowledge points. Yang et al. [33] proposed a Graph-based Interaction model for Knowledge Tracing (GIKT). It modeled the interactions between exercises and knowledge points. And integrated exercise-knowledge point correlation via embedded propagation using graph convolutional networks (GCN). However, graph-based KT models need to presuppose the existence of data correlations and limited scope of use.

Based on the above KT models analysis, this paper provides an overview of relevant research models, as show in Table 1.

**Table 1:** Summary of relevant research models

Models	Paper numbers	Advantages	Limitation
Traditional knowledge tracing models	[2,14–17]	Highly interpretable	Static diagnostics, multiple knowledge points difficult to interpret
Sequence modeling KT models	[3,18–21]	Making the most of test data and improving forecasting accuracy	Single dimension data analysis. Lack of consideration of other factors affecting students’ knowledge state
Attentive KT models	[4,22–26]	Considering how different historical interactions affect students differently	Model construction is complex and requires large amounts of data for training
Text-Aware KT models	[27–30]	Full consideration of exercise data, with different levels of difficulty associated with text content	Poor harmonization of datasets and difficulties in text tagging
Graph-Based KT models	[31–33]	Considering the spatial relationship of knowledge-knowledge. Automatic acquisition of edge weights and updating of cognitive abilities	The existence of data correlation needs to be preassumed and limited scope of use

In summary, most of existing KT models have been studied mainly for online education platforms. And it is difficult to accurately characterize the knowledge state of students’ in complex smart classroom. The reality that knowledge state is often complex and ambiguous. Students are susceptible to the influence of students around them in smart classroom, so it is challenging to accurately characterize students’ knowledge state. In summary, This paper is based on classroom network structure learning engagement and knowledge-exercise data. Using parallel temporal attention LSTM to track students’ accumulation, forgetting and learning basis of knowledge points. Mining the correlation properties of student learning with knowledge–knowledge, previous-Learning engagement and post learning engagement. Achieving accurate knowledge diagnosis.

## Relevant definitions

### Marker Definition

According to the course data, the set of learners of a course is noted as $U=\{u_{1},u_{2},...,u_{p}\}$, here *p* is the number of learners. The set of knowledge points of the course is represented as $K=\{kp_{l},kp_{2},...,kp_{k}\}$. with a total of *k* knowledge points. The exercise set for the course is represented as $E=\{e_{1},e_{2},...,e_{m}\}$. with a total of *m* test questions. The containment relationship between questions and knowledge points is represented as matrix $Q=(q_{mk})\in {\mathbb{R}}^{m\times \kappa }$. where $q_{mk}\in \{0,1\}$, represented as whether question *e_m_* contains knowledge point *kp_k_* or not. The test of learner *u* is recorded as the vector $X=\{x^{1},x^{2},...,x^{m}\}\in {\mathbb{R}}^{m}$.

### Modeling learning engagement based on classroom network structure

#### Smart classroom learning engagement.

Students’ heads-up attention state corresponds to their learning engagement in smart classrooms. A higher heads-up rate indicates student focus in class. Therefore, through video monitoring systems that monitor students’ heads-up rate and correlate them with learning engagement in smart classroom. It can allows teachers to better observe and assess students’ behaviors and understand their learning state. According to Shou et al. [34] proposed to analyze the learner’s head pose assessment in the smart classroom video. To obtain the heads-up rate of learner $u_{i}$ during the time duration of learning *k* knowledge point as Eq. (1).


$$\begin{array}{ccc} L_{u_{i}k}=\frac{t}{m} & & \end{array}$$ (1)

Here, *m* represents the number of image frames extracted within the knowledge point, and *t* represents the number of heads-up frames. In this paper, the heads-up rate within the knowledge point duration obtained from the learner’s head pose assessment model. It is used as the learning engagement in the smart classroom.

#### Classroom network structure learning engagement.

**Fig 1:**
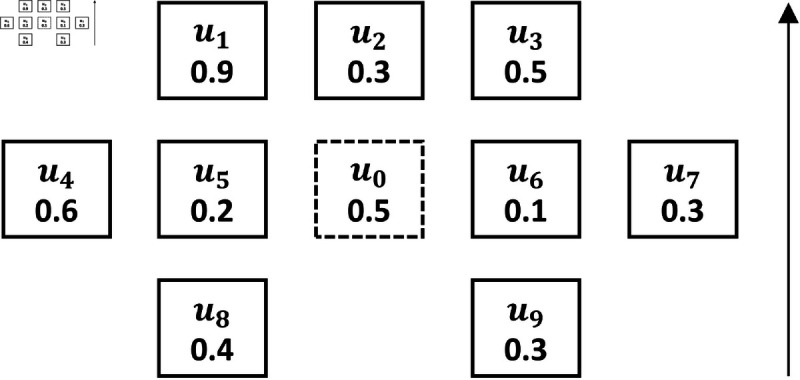
Effective distance for near-neighbor relationships.

In smart classroom, students with seats in close neighborhood are more likely to influence each other [35]. In this paper, the nearest-neighbor nodes around a node are selected as learning influence factors based on the Nearest-Neighbor Effective Distance Criterion (NEDC) [13]. The solid box nodes in Fig 1 indicate the NEDC range near-neighbor nodes that have a learning influence on node $u_{0}$.

**Fig 2:**
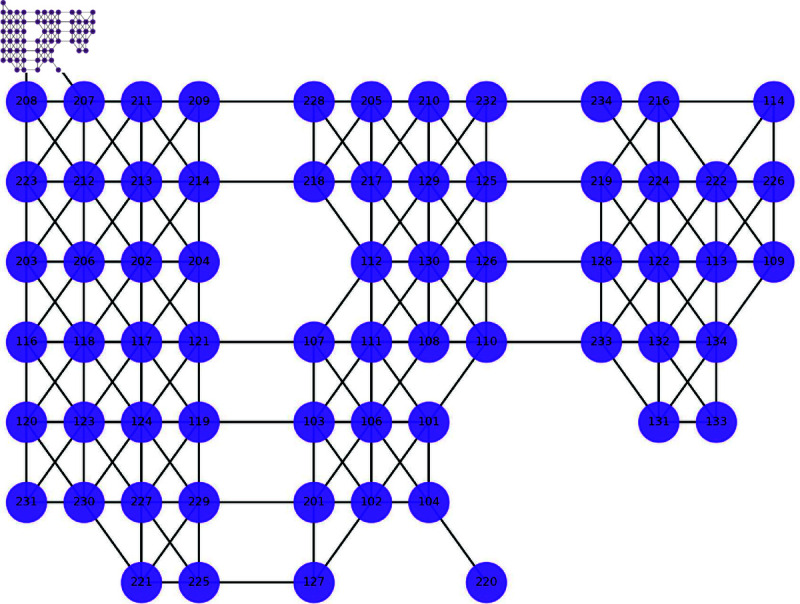
Single classroom seating relationship network $G_{UR}$.

The seat relationship data is used to construct the entitled and undirected seat relationship network $G_{UR}=\left(U,E_{UR}\right)$ that shown in Fig 2. Here *U* represents the set of student nodes and $E_{UR}$ represents the set of edges. This is expressed as follows:


$$\begin{array}{ccc} \{\begin{array}{c} U=\left\{u_{0},u_{1},\ldots ,u_{nt}\right\}\text{,}nt=student\text{ on }attendance\;\\ E_{UR}=\left\{\left(u_{i},u_{j}\right),d_{u_{i},u_{j}}|u_{i},u_{j}\in NEDCrange\right\}\; \end{array} & & \end{array}$$ (2)

Here, $d_{u_{i},u_{j}}$ represents the weight corresponding to edge $\left(u_{i},u_{j}\right)$. This is specified as the inverse


$$\frac{1}{\sqrt{{\left(x_{i}-x_{j}\right)}^{2}+{\left(y_{i}-y_{j}\right)}^{2}}}$$

of the Euclidean distance between the $u_{i}$ and $u_{j}$ seat coordinates $\left(x_{i},y_{i}),(x_{j},y_{j}\right)$.

Taking learner $u_{0}$ as an example to calculate its learning engagement based on the classroom network structure as shown in Fig 1. According to Fig 1 to obtain the nodes and edge weight set within the influence of $u_{0}$ immediate neighbors as $E_{u_{0}R}$.


$$\begin{array}{ccc} E_{u_{0}R}=\left\{\left(u_{0},u_{j}\right),d_{u_{0},u_{j}}|u_{j}\in u_{0-NEDC},n=mumberofNEDCranges,j\in n\right\} & & \end{array}$$ (3)

learner $u_{0}$ combines the learning engagement $L_{u_{j}k}$ and node-to-node weight $d_{u_{0},u_{j}}$ of the nodes within NEDC range the knowledge point *k* lecture time. To obtain the learning engagement $Net_{u_{0}}^{k}$ of learner $u_{0}$ based on the classroom network structure.


$$\begin{array}{ccc} Net_{u_{0}}^{k}=\frac{1}{n+1}\left[\overset{n}{\underset{\begin{array}{c} j=1\\ u_{j}\in E_{u_{0}R} \end{array}}{\sum }}\left(L_{u_{j}k}∙d_{u_{0},u_{j}}\right)+L_{u_{0}k}\right] & & \end{array}$$ (4)

Here, *n* represents the number of nearest neighbor influencing nodes and $L_{u_{o}k}$ represents the own learning engagement.

## CL-PTKT model

**Fig 3:**
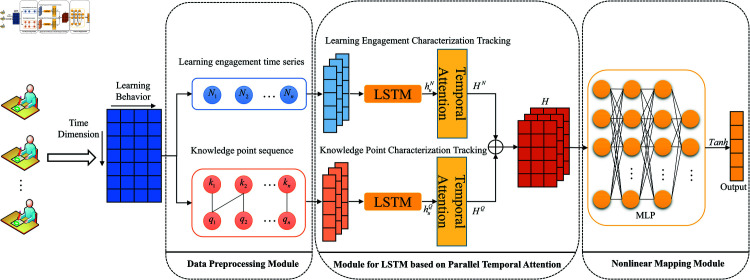
CL-PTKT model architecture.

According to the above definition, the CL-PTKT model consists of the following main steps: First, according to the actual conditions of the dataset, the classroom network structure learning engagement and knowledge point test sequences were feature coded separately. Second, the feature encoding vectors are inputted into a parallel temporal attention LSTM network to track the accumulation of knowledge points, forgetting and learning bases, so as to obtain synchronized feature tensor and fuse them. Finally, the fused feature tensor is inputted into the multilayer perceptron network(MLP) for nonlinear mapping to obtain the knowledge diagnosis of students’ temporal knowledge points.The CL-PTKT model architecture is shown in Fig 3. (This paper’s algorithm source code and all datasets available at: https://github.com/LL-stars/Code-Dataset).

### Feature tracking based on temporal attention LSTM

#### LSTM feature tracking.

**Fig 4:**
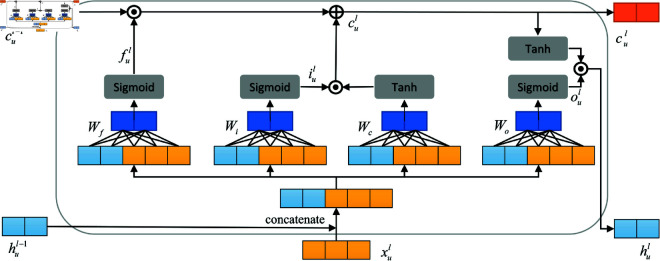
LSTM structure.

LSTM is mainly used to model sequence of time-series data, which can effectively mine the temporal and semantic information in the data [36]. The LSTM structure is shown in Fig 4.

In this paper, the student’s question records are divided into sequences with a fixed length *l* as the interval. If the student’s question records are less than the length *l*, then complement 0. So as to get the set *X* of all students’ question sequences. LSTM is used to capture the long dependencies in the data of test questions *X* taken by the learners. As well as the long dependencies in the classroom network structure learning engagement *Net* within the learning hours of the knowledge points involved in the test questions. Here, *X* is shown in Eq. (5) and *Net* is shown in Eq. (6).


$$\begin{array}{ccc} X={\left[\begin{array}{c} x^{1}\\ x^{2}\\ \vdots \\ x^{N} \end{array}\right]}_{N\times 100\times k} & & \end{array}$$ (5)

$$\begin{array}{ccc} Net={\left[\begin{array}{c} net^{1}\\ net^{2}\\ \vdots \\ net^{N} \end{array}\right]}_{N\times 100\times 1} & & \end{array}$$ (6)

Before inputting into the LSTM, the knowledge point test questions and the classroom network structure learning engagement sequence data are feature coded separately. Here, the one-hot encoding of a particular knowledge point test question sequence data is *x*.


$$\begin{array}{ccc} x=\left(\begin{array}{c} e^{1}\\ e^{2}\\ \vdots \\ e^{l} \end{array}\right)={\left[\begin{array}{ccccc} 0 & 1 & \cdots \; & 0 & 0\\ 1 & 0 & \cdots \; & 1 & 1\\ \vdots & \vdots & \ddots & \vdots & \vdots \\ 1 & 1 & \cdots \; & 0 & 0 \end{array}\right]}_{l\times (k\times 2)} & & \end{array}$$ (7)

Here, $e^{l}$ represents the vector of test question-knowledge point relationships for the test question with serial number *l* taken by the learner. Here, 0 represents that the test question does not involve the knowledge point corresponding to the subscript. 1 represents that it involves the knowledge point corresponding to the subscript.

The sequence of knowledge point test questions corresponds to the classroom network structure learning engagement input coded as *net*:


$$\begin{array}{ccc} net=\left(\begin{array}{c} ne^{1}\\ ne^{2}\\ \vdots \\ ne^{l} \end{array}\right)={\left[\begin{array}{c} 0.6\\ 0.7\\ \vdots \\ 0.5 \end{array}\right]}_{l\times 1} & & \end{array}$$ (8)

Here, $ne^{l}$ represents the classroom network structure learning engagement. It contains test questions *l* involved all the knowledge points of the classroom network structure learning engagement.

According to the feature coding of the learners’ temporal test data. And feature coding of classroom network structure learning engagement within the learning hours of the knowledge point corresponding to the test data. The input data are linearly transformed using LSTM. And then the results are passed to the activation function and mapped to the fixed range (0,1) to control the flow of information. Three gating units, the forgetting gate $f_{u}^{\;\;l}$, the input gate $i_{u}^{l}$ and the output gate $o_{u}^{l}$, realize the functions of selectively forgetting the information of the previous moment, selectively memorizing the information of the current moment and selecting the information as the output of the current moment. The calculation is shown in Eq 9:


$$\begin{array}{ccc} \{\begin{array}{c} f_{u}^{l}=Sigmoid(W_{f}x_{u}^{l}+W_{f}h_{u}^{l-1}+b_{f})\;\\ i_{u}^{l}=Sigmoid(W_{i}x_{u}^{l}+W_{i}h_{u}^{l-1}+b_{i})\;\\ o_{u}^{l}=Sigmoid(W_{o}x_{u}^{l}+W_{o}h_{u}^{l-1}+b_{o})\; \end{array} & & \end{array}$$ (9)

Similarly the input data $x_{u}^{l}$ and $h_{u}^{l-1}$ are linearly transformed. The result is passed to the *Tanh* function. The final result is the information $a_{u}^{l}$ to be input into the current LSTM cell’s state vector $c_{u}^{l}$. The information $a_{u}^{l}$ to be input into the current LSTM cell is shown in Eq. (10).


$$\begin{array}{ccc} a_{u}^{l}=Tanh(W_{c}x_{u}^{l}+W_{c}h_{u}^{l-1}+b_{c}) & & \end{array}$$ (10)

The state $c_{u}^{l-1}$ of the previous moment and the pending input information $a_{u}^{l}$ of the current moment are selectively memorized and inputted, respectively, with the results of the two being summed to obtain the memory cell vector $c_{u}^{l}$ to maintain the memory of the LSTM cell in real time. Eventually, the sequence state vector $h_{u}^{l}$ is obtained by multiplying the output gate output vector $o_{u}^{l}$ and the memory cell vector $c_{u}^{l}$ after the activation function *Tanh*. This is shown in Eq. (11).


$$\begin{array}{ccc} \begin{array}{c} c^{l}=f^{l}\cdot c^{l-1}+i^{l}\cdot a^{l}\\ h^{l}=o^{l}\cdot Tanh(c^{l}) \end{array} & & \end{array}$$ (11)

The feature tensor $h_{Q}$ and $h_{N}$ are obtained by tracking both the knowledge point test data features and the classroom network structure learning engagement features using parallel temporal attention LSTM networks.

#### Temporal attention mechanismt.

The attention mechanism in deep learning facilitates the neural network model to focus on the information more critical to the current task in the input information. It can effectively reduce the problem of the LSTM’s loss of capturing the long sequence information and the shortage of extracting the short sequence feature information. So as to improve the model’s performance and generalization ability [37]. Therefore, this paper proposed the temporal attention mechanism to reassign the weights to the temporal knowledge points and learning engagement feature information from LSTM output. It can better extract the feature information related to the current knowledge points and improve the model prediction accuracy. The specific calculation of the temporal attention mechanism is shown in Eq. (12).


$$\begin{array}{ccc} \{\begin{array}{c} H^{Q}=\text{Relu6}(h^{Q}∙\text{Softmax}(Tanh(h^{Q}\times W_{Q}),dim=1))\;\\ H^{N}=\text{Relu6}(h^{N}∙\text{Softmax}(Tanh(h^{N}\times W_{N}),dim=1))\; \end{array} & & \end{array}$$ (12)

**Fig 5:**
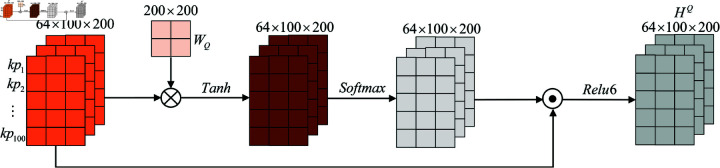
Visualization of the temporal attention mechanism for the weight reassignment of the knowledge point test feature information.

Here, the visualization of the temporal attention mechanism to reassign weights to the temporal knowledge point test data feature information is shown in Fig 5.

**Fig 6:**
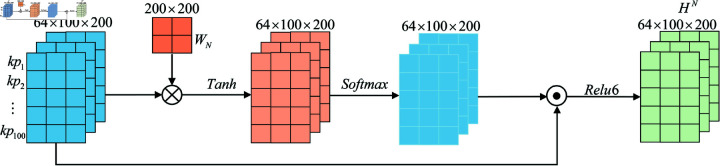
Visualization of the temporal attention mechanism for the weight reassignment of the classroom network structure learning engagement feature information.

Similarly, the visualization of the temporal attention mechanism to reassign weights to the temporal classroom network structure learning engagement feature information is shown in Fig 6.

Eventually the features $H^{Q}$ and $H^{N}$ of the parallel temporal attention LSTM outputs are added to fuse them for obtaining the feature tensor *h*. The calculation is shown in Eq. (13).


$$\begin{array}{ccc} H=H^{Q}+H^{N} & & \end{array}$$ (13)

### MLP-based nonlinear mapping

**Fig 7:**
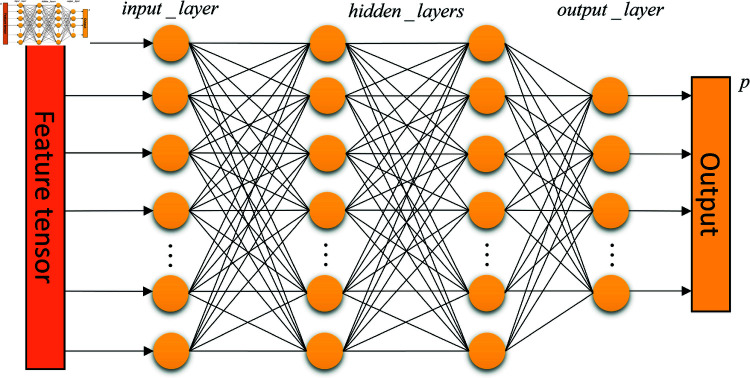
MLP network.

MLP networks have nonlinear fitting ability to capture complex data patterns, and the main role is to map the input tensor nonlinearly. To extract the correlation information between features and map it to the output space [38]. The MLP network used in this paper is shown in Fig 7.

The final output of the fusion tensor through the MLP network is calculated as shown in Eq.


$$\begin{array}{ccc} p=Tanh\left(MLP(H)\right) & & \end{array}$$ (14)

Here, *p* represents the model prediction, and *H* represents the feature fusion tensor of the output of the parallel temporal attention LSTM.

The output layer takes the learners’ real answer as the target. It iteratively updates the model parameters using the cross-entropy loss function. As shown in Eq. (15):


Loss=−∑xlj∈Xplj∈P [xlj log ⁡  (plj)+ (1−xlj) log ⁡  (1−plj)] (15)

Here, *xl_j_* represents the actual answer to knowledge point *j* involved in the *l* exercise, and *pl_j_* represents the predicted value of the model prediction for the knowledge point *j* involved in exercise *l*.

Algorithm 1 illustrates the basic steps of CL-PTKT:



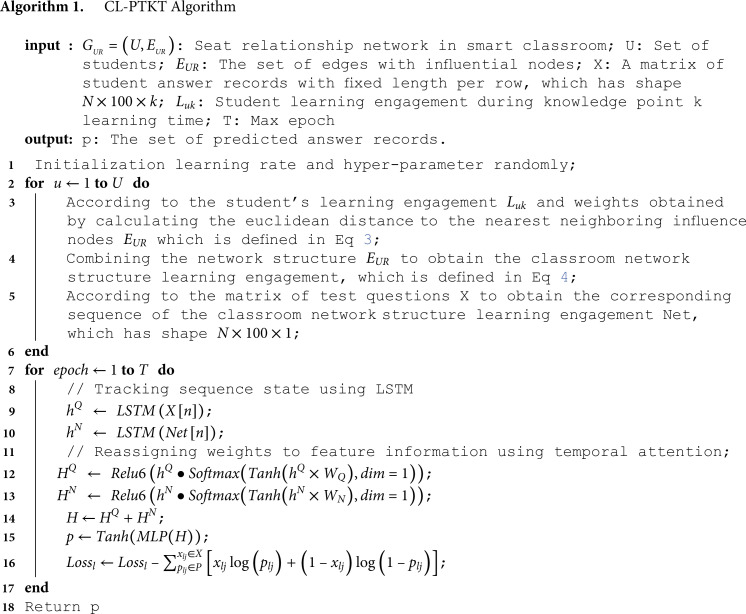



## Experimental results and analysis

### Dataset

To validate the performance of the proposed CL-PTKT model, the experiments are conducted on four real datasets. They are Assistment0910, Assistment2017 [39], Eedi [40] and SCD(Smart Classroom Dataset) datasets, respectively.

The ASSISTment0910 dataset was collected by the ASSISTments online learning system during the 2009-2010 school year and contains records of unanswered and repeated exercises. It contains 4,217 students, 401,756 knowledge interactions, 123 knowledge points and 26,688 exercises.

The ASSISTment2017 dataset was collected by the ASSISTments online learning system during the 2017–2018 academic year and contains records of unanswered and repeated exercises. It contains 1,709 students, 942,816 knowledge interactions, 102 knowledge points and 3,162 exercises.

The Eedi dataset was released by the NeuralPS2020 Education Challenge and consists of four task datasets. In this paper, Task 3 and Task 4 parts of this dataset are selected for the knowledge tracing task. It contains 4,918 students, 1,048,575 knowledge interactions, and 948 exercises. Since there is no official information on which knowledge points are included in the exercises, each exercise is treated as a knowledge point in this paper.

The SCD dataset was a smart classroom dataset consisting of historical behavioral data of students attending the C Programming course in the year 2022. It contains 8 classroom videos teaching the process of the knowledge points, 58 students, 11,312 knowledge interactions data, 37 knowledge points and 45 exercises.

Because the Assistment0910, Assistment2017, and Eedi datasets do not contain information about the classroom network of the learner. They are supplemented with a value of 0 in these three datasets to ensure the wholeness of the model and to minimize the impact on the datasets. The statistics of the four datasets are shown in Table 2.

**Table 2:** Dataset statistics

Dataset	Assistment0910	Assistment2017	Eedi	SCD
#learners	4,217	1,709	4,918	58
#skills	123	102	948	37
#num of excerises	26,688	3,162	948	45
#interactions	401,756	942,816	1,048,575	11,312
#average-length	95.3	551.7	213.2	195
#engage-data	No	No	No	Yes

### Evaluation indicators and baseline model

In order to evaluate the performance of the CL-PTKT model proposed in this paper, AUC, ACC, MAE and RMSE are used as evaluation metrics, which are also commonly used in knowledge tracing models.

In order to validate the advantages of the CL-PTKT model. Five baseline models are used for comparison experiments with the CL-PTKT model on four datasets, as shown in Table 3.

**Table 3:** Baseline model

Baselines	Description
DKT [18]	Tracking students’ knowledge state by using Recurrent Neural Networks (RNN).
DKVMN [19]	Inspired by memory-enhanced neural networks, a static and dynamic matrix is constructed to store and update with all the concepts and students’ knowledge states, respectively.
AKT [23]	Attention tracing models using exponential decay and context-aware relative distance measures
QRCDM [3]	A cognitive diagnostic model based on explicit correlations between test questions and knowledge concepts. And implicit correlations between test questions and unrelated knowledge concepts.
CL4KT-FoLiBi [20]	A knowledge tracing model Incorporating Forgetting-aware Linear Bias (FoLiBi) and comparative learning frameworks.

### Experimental environment and hyper-parameter settings

The experimental environment of this paper is shown in Table 4.

**Table 4:** Experimental environment

Experimental environment	Environment configuration
Operating systems	Linux
CPU	Intel(R) Xeon(R) Gold 6330H
Video Cards	GeForce RTX 3090
RAM	32 GB
ROM	1 T SSD
Programming Languages	Python 3.8
Framework	Pytorch

In the CL-PTKT model, the learning rate is set to 0.001. The length of the input feature sequence *l* is set to 100. The number of one-sided LSTM layers is set to 1. The embedding dimension of the hidden layer is set to 200. The batch size of the model is set to 64, and the gradient descent optimization is carried out using the Adam optimizer.

### Results

**Fig 8:**
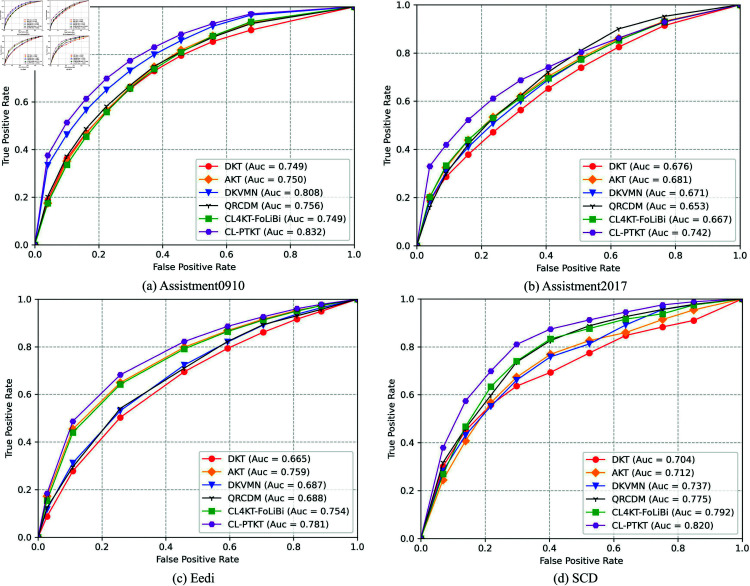
ROC curves for the CL-PTKT model and the baseline model on the dataset. (a–d) represent the Assistment0910, Assistment2017, Eedi, and SCD datasets, respectively.

The ROC curves for each model on the Assistment0910, Assistment2017, Eedi and SCD datasets as shown in Fig 8. The ROC curves for each model confronting the e-learning platform dataset using only the knowledge point test question data as shown in Fig 8a–c. The analysis shows that the AUC value of the CL-PTKT model is better than that of the baseline model. Even if they are online platform datasets using only the knowledge point test data. Fig 8d represents the diagnostic performance of the model on the SCD dataset. The CL-PTKT model that incorporates learners’ classroom network structured learning engagement exhibits better performance.

The performance metrics of the CL-PTKT model on the Assistment0910, Assistment2017, Eedi, and SCD datasets with other baseline models are shown in Table 5 and analyzed as follows:

**Table 5:** Performance metrics of all models on the dataset

Dataset	Model	AUC	ACC	MAE	RMSE
Assistment0910	DKT	0.749	0.734	0.298	0.517
	AKT	0.750	0.715	0.285	0.452
	DKVMN	*0.808*	*0.761*	*0.238*	0.488
	QRCDM	0.756	0.727	0.272	0.522
	CL4KT-FoLiBi	0.749	0.702	0.298	*0.449*
	**CL-PTKT**	***0.832***	***0.792***	***0.201***	***0.448***
Assistment2017	DKT	0.676	*0.668*	*0.332*	0.576
	AKT	*0.681*	0.656	0.343	***0.468***
	DKVMN	0.656	0.666	0.334	0.578
	QRCDM	0.653	0.623	0.376	0.613
	CL4KT-FoLiBi	0.667	0.656	0.344	*0.471*
	**CL-PTKT**	***0.742***	***0.720***	***0.280***	0.529
**Eedi**	DKT	0.665	0.633	0.336	0.605
	AKT	*0.759*	*0.694*	*0.306*	***0.445***
	DKVMN	0.687	0.637	0.363	0.603
	QRCDM	0.687	0.662	0.439	0.471
	CL4KT-FoLiBi	0.754	0.688	0.311	*0.449*
	**CL-PTKT**	***0.781***	***0.712***	***0.287***	0.536
**SCD**	DKT	0.704	0.724	0.276	0.525
	AKT	0.712	*0.809*	0.276	0.517
	DKVMN	0.737	0.789	0.264	*0.466*
	QRCDM	0.775	0.780	0.220	0.470
	CL4KT-FoLiBi	*0.792*	***0.811***	*0.202*	0.468
	**CL-PTKT**	***0.820***	0.800	***0.199***	***0.447***

Bold-italics denotes the optimal result. Italics denotes the suboptimal result.

(1) The method proposed in this paper achieves the best performance in most of the evaluation metrics. In the SCD dataset, most of the evaluation metrics are better than the baseline model, and that demonstrates the effectiveness of integrating the classroom network structure learning engagement.
(2) Comparing with the RNN-based DKT model and the DKVMN based on different activation functions to compute the accumulation and forgetting of knowledge points. CL-PTKT has a remarkable improvement. It proves the superiority of computing accumulation and forgetting based on the dynamic control gates of LSTM.
(3) AKT mainly uses monotonic attention mechanism for contextual feature extraction, and QRCDM mainly uses the feature extraction method of *α* cross-validation idea. CL-PTKT enhances the feature extraction by using the temporal attention mechanism. The results are better than those of AKT, QRCDM. It proves that the temporal attention mechanism can better pay attention to the important feature information. As well as enhance the model prediction accuracy.
(4) CL4KT-FoLiBi embeds the forgetting linear deviation mechanism to simulate students’ forgetting behavior, but ignores the fact that in reality the students’ knowledge process is ambiguous and complex. The CL-PTKT takes the aspect of enhanced feature extraction and achieves better results.
(5) According to Table 5, the CL-PTKT model shows superior performance in all three online learning platform datasets and one smart classroom dataset. In this case, the distribution and number of the four datasets are different, as to prove that the CL-PTKT model has stronger robustness.
(6) The RMSE values on the Assistment2017 dataset, the CL-PTKT model underperforms comparing with some baseline model. After experimentation, because of the long average length of student test sequences in the Assistment2017 dataset. The poor performance of the RMSE value CL-PTKT on the Eedi dataset. This is because the dataset contains many knowledge points.

### Ablation experiment

#### Effects of temporal attention mechanisms.

**Fig 9:**
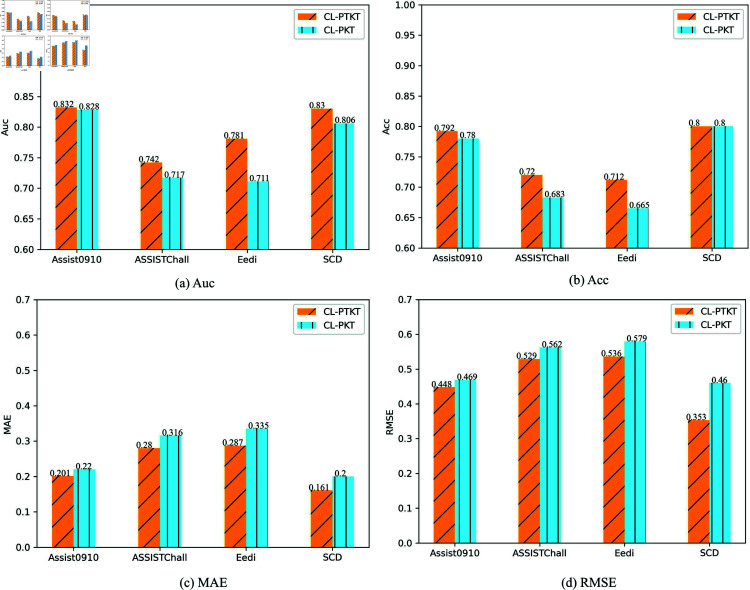
Performance metrics of CL-PTKT and CL-PKT models on four datasets. (a–d) represent the Auc, Acc, MAE, and RMSE values of the model with or without the temporal attention mechanism, respectively.

In order to verify the advancement of the proposed temporal attention mechanism. It can enhance the model to capture learner’s feature information and improve the model’s prediction ability. This paper conducts the ablation experiments of the temporal attention mechanism on four datasets. The CL-PKT represents the model without the addition of the temporal attention mechanism. The results of the experiments are shown in Fig 9.

According to Fig 9 and the analysis of the experimental results, CL-PTKT outperforms CL-PKT on all four datasets. It proves that CL-PTKT has better stability and robustness.

In summary, adding the temporal attention mechanism to LSTM can enhance the feature extraction ability of the model. It effectively improves the stability and prediction ability of the model.

#### Classroom network structure learning engagement ablation experiment.

**Fig 10:**
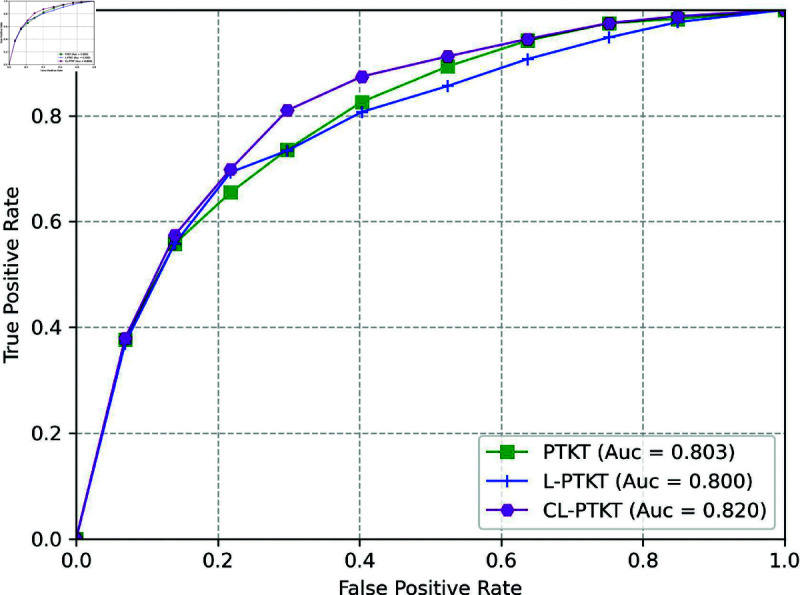
Comparison of ROC curves for ablation experiments on the SCD dataset for the CL-PTKT model.

In order to illustrate that in smart classrooms with complex environments, fusing learners’ classroom network structure learning engagement model(CL-PTKT) has better predictive performance. This paper conducts ablation experiments on the SCD dataset. The results of the experiment are shown in Fig 10. Here, PTKT represents without integrating the learner’s classroom network structure learning engagement, and L-PTKT represents integrating its own learning engagement model.

**Table 6:** Performance metrics of CL-PTKT model based on different learning engagement

Dataset	Model	AUC	ACC	MAE	RMSE
	PTKT	*0.803*	*0.787*	*0.212*	0.461
SCD	L-PTKT	0.800	0.787	0.212	*0.460*
	**CL-PTKT**	***0.820***	***0.800***	***0.199***	***0.457***

Bold-italics denotes the optimal result. Italics denotes the suboptimal result.

**Table 7:** Comparison of the accuracy of the PTKT, L-PTKT and CL-PTKT modelss

ID	PTKT (%)	L-PTKT (%)	CL-PTKT (%)
108	75.9	77.0	77.3
112	88.3	89.2	89.8
116	91.1	89.7	91.6
206	87.6	87.5	90.4
207	73.5	75.9	76.5
212	80.4	76.8	81.7

The performance metrics of using different classroom network structure learning engagement models on the SCD dataset are shown in Table 6.

Meanwhile, this paper conducts experimental validation on some students within 37 knowledge point lecture time. The results of the comparison of the accuracy of the knowledge state diagnosis results output from the PTKT, L-PTKT and CL-PTKT models are shown in Table 7.

From the analysis of Fig 10, Tables 6 and 7, the performance of the CL-PTKT model incorporating classroom network features outperformed that without the incorporation of classroom network features. It also outperforms the model that incorporates the learner’s own learning engagement features. The overall results indicate that in smart classroom with multiple learners, a single dimension of test question data cannot accurately predict student performance. In addition, students’ learning engagement does not only depend on themselves, but also the learners’ learning engagement will be influenced by the nearest-neighbor nodes based on the seating relationship network. These influencing factors play a direct role in learners’ grasp of knowledge points during the learning process. It is ultimately reflected in the results of the knowledge point test.

### Analysis of results

In order to verify the effectiveness of the model’s predicted outputs for knowledge state diagnosis. This paper uses learners’ classroom test scores as targets on the SCD dataset for classification validation. this paper uses learners’ classroom test scores as targets on the SCD dataset for classification validation. Some of this student knowledge state diagnostic results from the predicted output of the CL-PTKT model are shown in Table 8.

**Table 8:** Diagnostic results of students’ knowledge state within each knowledge point’s lecture time

ID	KP1	KP2	KP3	...	KP36	KP37
108	0.711	0.819	0.387	...	0.744	0.799
112	0.695	0.831	0.604	...	0.847	0.801
116	0.716	0.620	0.666	...	0.873	0.892
206	0.695	0.926	0.810	...	0.811	0.758
207	0.717	0.705	0.478	...	0.819	0.726
212	0.510	0.560	0.687	...	0.851	0.406

In order to assess the reliability of the resulting knowledge state diagnostic results, the cognitive state levels of the pyramid proposed by Bloom [41] were relied upon (memorization, comprehension, application, analysis, evaluation, and innovation). According to the correlation between test scores and cognitive levels proposed by [42–46] et al. The learners’ knowledge diagnostic results are separated into intervals by the duration of the knowledge points learned in the course. The results are categorized as Memory ([0,40), F), Comprehension ([40,65), E), Application ([65,80), D), Analysis ([80,90), C), Evaluation ([90,97), B), and Innovation ([97,100], A), as shown in Table 9.

**Table 9:** Diagnostic results of students’ knowledge state within each knowledge point’s lecture time

ID	KP1	KP2	KP3	...	KP36	KP37
108	D	C	F	...	D	D
112	D	C	E	...	C	C
116	D	E	D	...	C	C
206	D	B	C	...	C	D
207	D	D	E	...	C	D
212	E	E	D	...	C	E

In the end, the results of Table 8 are combined to analyze the knowledge diagnostic results of these six students within these 37 lecture times of knowledge points. Also comparing each student’s performance on the classroom final test, as shown in Table 10.

**Table 10:** Students’ comprehensive cognitive state and final examination scores

ID	knowledge diagnostic results	Test performance	Test grade
108	D	79	D
112	C	90	B
116	D	73	D
206	C	82	C
207	D	67	D
212	E	51	E

Classroom tests are effective in assessing students’ cognitive state. Cognitive state affect students’ learning outcomes. When students are in an environment of positive learning engagement, they are more aware of the knowledge points and their assessment scores are relatively higher. According to Table 9, the results obtained from the cognitive state diagnostic model proposed in this paper correspond to their test scores. The students with higher test scores are basically in a higher level of cognitive state. It indicates that the proposed cognitive state diagnostic model has good reliability.

### Discussion

Our proposed CL-PTKT model can be used as an auxiliary teaching module of the smart classroom teaching platform to help teachers improve the teaching process design and improve teaching efficiency. By observing the students’ knowledge tracking results predicted by the model, teachers can have a deeper understanding of students’ current knowledge status, and then intervene in time. Students can know what knowledge they have mastered well enough according to the knowledge tracking results, so as to clarify their learning objectives and improve their academic performance.

The above experimental results show that in the smart classroom scenario, the integration of learning engagement based on classroom network structure can effectively improve the accuracy of the model in predicting students ’knowledge state. Similarly, in the field of online education, students ’learning engagement can be obtained by collecting information such as the frequency and duration of interaction between students and knowledge points, so as to predict students ’knowledge status more accurately. Therefore, this model can also be considered to be applied to online education platforms to track students’ knowledge status, and provide stronger interpretability for teaching recommendation systems according to the knowledge tracking results.

## Conclusions and future work

In this paper, a classroom network structure learning engagement and parallel temporal attention LSTM based knowledge tracing model (CL-PTKT). Aiming to address the inability to accurately characterize student knowledge state in complex smart classroom, and the problem that existing KT models only consider historical test behavior data of students on online educational platforms. The CL-PTKT model is effective in characterizing students’ knowledge state even with limited interaction data in smart classroom, as well as shows superior performance even in the face of large datasets online. The experimental results show that the prediction accuracy of the learning engagement degree model(CL-PTKT) of the fusion classroom network structure is better than the benchmarking model(PTKT, L-PTKT). Moreover, the use of parallel temporal attention LSTM helps to enhance the extraction of feature information, which further improves the accuracy of model prediction. Extensive experiments are conducted on four real datasets. It shows that the CL-PTKT model exhibited better diagnostic performance compared to five baseline models (DKT, DKVMN, AKT, QRCDM, CL4KT-FoLiBi) under different conditions.

The CL-PTKT model can effectively address the problem of diagnosing students’ knowledge state in complex smart classrooms, and enhance the design of the education platform system. Characterizing students’ knowledge state by incorporating classroom network structure learning engagement. It can help teachers make timely adjustments to student seating during the teaching process, and appropriate interventions. Improving students’ attitudes towards learning and learning engagement. This promotes effective and enhanced student learning. This research can be applied to online platforms and oﬄine smart classroom platforms. but existing image processing techniques applied to the smart classroom in a large scene have problems such as the difficulty of recognizing students in the back row, therefore, the classroom network structure data in this study can not be fully automated, and it is necessary to add a manual proofreading link, which increases a certain amount of workload.

In future research, the effects of different classroom social relationships and network characteristics on student cognition will be considered, so as to achieve a more accurately characterize cognitive state and improve the predictive performance of the model. In addition, this paper plans to deploy the algorithm into embedded devices for smart classrooms. To provide effective support for teachers to make precise interventions on students during the teaching process. So as to continuously improve the students’ learning effect.
